# Reliability and validity of the Portuguese version of the Generalized Anxiety Disorder (GAD-7) scale

**DOI:** 10.1186/s12955-015-0244-2

**Published:** 2015-04-25

**Authors:** Tiago V Sousa, Vânia Viveiros, Maria V Chai, Filipe L Vicente, Gustavo Jesus, Maria J Carnot, Ana C Gordo, Pedro L Ferreira

**Affiliations:** Lisbon Psychiatric Hospital, Lisbon, Portugal; Medical Unit, Pfizer, Porto Salvo, Portugal; Centre for Health Studies and Research, University of Coimbra, Coimbra, Portugal; Faculty of Economics, University of Coimbra, Coimbra, Portugal

**Keywords:** Generalized anxiety disorder (GAD), Portuguese version, Validation

## Abstract

**Background:**

Generalized anxiety disorder has a strong impact on health-related quality of life. For this reason, it seems relevant to develop strategies allowing early diagnoses in order to promote appropriate treatments. The objective of this study was to culturally adapt and validate the GAD-7 for the Portuguese patients with generalized anxiety disorder.

**Methods:**

For the cultural adaptation of the Portuguese version of the GAD-7 scale we started with a previous translation made by Mapi Institute and decided to perform a clinical review followed by a cognitive debriefing with patients. Once piloted, this version was then tested in a larger sample for feasibility and reliability (1-week test-retest). Construct validity was assessed by the relationship between GAD-7 and socio-demographic and clinical variables. Its unidimensionality was tested by principal component factor analysis. Criterion validity was assessed by comparing GAD-7 scores with those obtained by HADS, and EQ-5D. STAI was mainly used as a screening indicator for patient inclusion.

**Results:**

GAD-7 was considered feasible with a mean completion time of 2.3 minutes and no major floor or ceiling effects. We found an excellent Cronbach’s alpha internal consistency score (0.880) and the test-retest and interclass correlation coefficients were also very good. Regarding the construct validity, younger patients, those with higher education, employed and without anxiety symptoms revealed lower GAD-7 scores, meaning better health. The unidimensionality of GAD-7 index was also confirmed by principal component factor analysis. At last, GAD-7 was significantly correlated with other health outcome indices and the classification levels created by it and by HADS showed to be dependent.

**Conclusion:**

The excellent metric properties confirmed the cultural adaptation and validity of GAD-7 into Portuguese population, allowing the clinicians an early detection and treatment of these patients.

## Introduction

Anxiety is the manifestation of an emotion, characterized by a physical and psychological discomfort described by individuals as a feeling of restlessness, nervousness and excessive concern [1-3]. Anxiety disorders are the most common psychiatric disorders in Europe, with an annual prevalence of 12% in the European adult population and a lifetime prevalence of 5%.

Generalized anxiety disorder (GAD) was included for the first time in the third edition of the DSM in 1980 [4]. Since then, its definition has been modified on subsequent DSM-III-R, DSM-IV and DSM-IV TR [5-8]. GAD is clearly distinguished from other anxiety and depression disorders in both DSM-IV-TR and ICD-10.

GAD is defined, by the text revision of the fourth edition of Diagnostic and Statistical Manual of Mental Disorders (*DSM-IV-TR*), as excessive anxiety and worry about several events or activities for most days during at least at 6-month period. The worry is difficult to control and is associated with somatic symptoms such as muscle tension, irritability, difficulty sleeping and restleness. The anxiety is distressing and produces impairment in important areas of the person’s life [9].

GAD lifetime prevalence was estimated on 2.8% in Europe [3,10,11]. The ratio of women to men with the disorder is about 2 to 1. This disorder has probably the highest comorbility with another mental disorder, such as depressive disorders, specific and social phobia, panic disorder and substance-related disorder [9].

Portugal has an annual mental illness prevalence of 22.9%, higher than other European countries. The question of how to explain such a high prevalence, different from what was found in other Southern European countries still remains to be answered. Could it be the exposure to more vulnerable and/or less protective factors in relation to mental illness, leading to a higher frequency of psychiatric disorder among the Portuguese population? If so, what is the nature and the role of the factors involved? Is it possible that these results can be explained by the existence in the Portuguese culture of specific patterns of perception and manifestation of emotional complaints leading to increased expression of symptoms that are the basis of the diagnosis of mental illness? At present, there are no definitive answers to these questions [12]. In addition, between 2008 and 2009, anxiety disorders were one of the most common disorders within the Portuguese population, with an annual prevalence of 16.5%. It was also found that 33.6% patients with a severe psychiatric disorder in Portugal did not receive any kind of treatment [12].

Although the exact cause of GAD cannot be specified, there are population groups at greater risk with high comorbidity [10]. The highest prevalence occurs in the 45–59 age group, and it was more common in women (7%) than in men (4%). Other important predictors include being separated, widowed or divorced, unemployed or housewife [13,14].

Several studies have suggested that GAD negatively impacts on activities of daily life and patients’ health-related quality of life (HRQoL), and results in the possibility of decreased lifetime work productivity, thereby having a significant economic burden [15-18]. The literature showed that the strong impact of GAD on HRQoL is greater than the one observed in major depression [19], seeming relevant the development of strategies allowing early diagnoses, in order to promote appropriate treatment.

Taking into account the evaluation of anxiety and the instruments internationally developed, surprisingly there were no instrument culturally adapted and appropriately validated for the Portuguese population [20]. Therefore the objective of this study was to culturally adapt and validate the GAD-7 scale to the European Portuguese population and to assess the psychometric properties of the adapted version in terms of feasibility, reliability and validity.

## Methods

### Description of GAD-7

The GAD-7 is a self-administered patient questionnaire normally used as a screening tool and as a severity measure for patients with generalized anxiety disorder [21,22]. It has a unidimensional structure matching the original structure of DSM-IV-TR diagnostic criteria with all items measuring the same concept and in the same direction. It is composed by seven items corresponding to symptoms based on the criteria for GAD in the Diagnostic and Statistical Manual of Mental Disorders [5-8] including (1) feeling nervous, anxious or on edge, (2) not being able to stop or control worrying, (3) worrying too much about different things, (4) trouble relaxing, (5) being so restless that it is hard to sit still, (6) becoming easily annoyed or irritable, and (7) feeling afraid as if something awful might happen. The time period for the measurement is the two previous weeks and, through a 4-point Likert scale from ‘not at all’ to ‘nearly every day’, it is asked how often the patient has been bothered by any of the presented problems.

The GAD-7 index is obtained by adding the scores from the questionnaire, after having assigned 0 to the least severe situation, 3 to the most severe one, and 1 and 2 to the intermediate ones. The cut off points 5, 10 and 15 allow us to classify the anxiety as none/normal (0–4), mild (5–9), moderate (10–14), and severe (15–21). In general, anyone who scores 8 or above can be considered as having significant anxiety symptoms [23].

### Linguistic and semantic equivalence

We based our study on the official Portuguese version copyrighted by Pfizer and already translated by Mapi Research Institute, a leading patient-centered research company. The linguistic validation of the GAD-7 into Portuguese aimed to obtain a conceptually equivalent version easily understood by patients. With the collaboration of the instrument’s developer, this rigorous methodology involved a process which comprised several steps: forward translations by two qualified translators, a reconciliated version, a translation by another qualified translator, and a cognitive debriefing on 5 healthy subjects [24,25].

However, to complete the linguistic and cultural adaptation we decided to perform a clinical review and a cognitive debriefing with patients. Both were considered a means to test the instrument’s content validity, *i.e.*, to evidence its suitability to the specific purpose. So, for a clinical review, we first asked a committee composed by both forward translators, six psychiatrists (authors), a medical advisor from Pfizer (author) and two other psychiatrists to clinically comment the Portuguese translation, taking into account the original one in English. Based on their remarks, we then made changes in the Portuguese version and performed a cognitive debriefing interviewing ten patients with the purpose of finding the presence of any problems of clarity, understandability and redundancy of the items. To assess the feasibility of GAD-7 we recorded the time taken by patients to fill the questionnaire, as well as the difficulties patients had in answering it. Missing values, floor and ceiling effects were also analysed.

### Study population

Once piloted, the Portuguese version was then tested for reliability and validity. For this second phase we recruited 100 patients and asked five psychiatrists from the Psychiatric Hospital Centre, in Lisbon, to give the questionnaires to patients. The sample size is considered an acceptable number for validation studies and for factor analysis [26]. Data were collected during a period of 5 months, starting in December 2012.

The study population consisted of all individuals who went for a consultation, in a consecutive way, having a diagnosis of GAD according to DSM-IV-TR criteria [8], and fulfilled the selection criteria outlined in the research protocol. The diagnosis was made by psychiatrists, based on clinical interview. The sample size was estimated taking into account the sensitivity of the GAD questionnaire. One hundred patients with GAD assure that a 95% confidence interval around a sensitivity of 0.90 is not greater than 0.05.

As an inclusion criteria we accepted patients of both genders, over 18 years old, able to understand and speak Portuguese, with known diagnosis of generalized anxiety disorder based on DSM-IV-TR [8], having anxiety symptoms with or without treatment. (score ≥ 20 points on STAI anxiety scale). Patients with health conditions that made them impossible to fill the scale without any help, with limited knowledge of the Portuguese language, illiterate, or under pharmacological treatment that interfere with their ability to understand and answer the questions, were excluded.

### Reliability

The reliability was tested by a 1-week test-retest. A sample of 30 patients were given the GAD-7 in those two different points in time and the Pearson, the item-total and the intraclasse correlation coefficients were computed. No clinical intervention occurred during this week. With the whole sample we also determined the internal consistency through the Cronbach’s alpha coefficient [27].

### Validity

In what concerns the validity tests, other official Portuguese validated versions of measurement instruments were implemented, namely, the self-administered generic quality of life instrument EuroQoL EQ-5D [28-30], the Hospital Anxiety and Depression Scale (HADS) [31,32] and the State-Trait Anxiety Inventory (STAI) [33], in their validated Portuguese versions. For the characterization of target population we also collected socio-demographic data (gender, age, educational level, family status and employment status) and some clinical data (clinical background, psychiatric and physical symptoms). The data were collected by psychiatrists.

The generic EQ-5D instrument was originally developed in the University of York, UK, and allows us to measure the global value that each individual assigns to his/her health status. It also yields to the construction of the utility indicator QALY (Quality-Adjusted Life Years) used on clinical and policy decision-making [28]. The dimensions measured by this instrument’s descriptive system are (i) mobility, (ii) self-care, (iii) usual activities, (iv) pain/discomfort, and (v) anxiety/depression. Each dimension is scored in a 3-item severity scale and an econometric algorithm produces an index ranging from −0.59 to 1.00 (negative scores meaning health states perceived as worse than death) and respecting the value set that society assign to each measured health status. EQ-5D also includes a visual analogue scale (VAS) designed to look like a vertical thermometer, ranging from 0, meaning the worst imaginable health state, to 100, meaning the best imaginable health state.

HADS aims to determine the levels of anxiety and depression that a patient is experiencing. It is composed by seven items relate to anxiety and seven other items related to depression. An important point that distinguishes HADS from other scales is that, to prevent the interference of somatic disorders on the scale score, all the symptoms of anxiety or depression related to physical diseases were deleted. Each item is scored from 0 to 3 and the maximum total score is 21 for each subscale. Also, again for each subscale, the authors proposed a cut-off point such that a score smaller than 9 corresponds to the absence of symptom and a score equal to 9 or higher corresponds to the presence of the symptom.

STAI is a self-reported measure that distinguishes between temporary condition of state anxiety and the longstanding quality of trait anxiety. It takes about 10 minutes to be filled and consists of two subscales, each of them containing 20 items: (i) the S-Anxiety to evaluate the current state of anxiety, and (ii) the T-Anxiety to evaluate the relatively stable aspects of “anxiety proneness” (trait). For each of these subscales the scores are added, although some of them need to be reversed, the total scores range from 20 and 80, where a high score indicates greater anxiety.

To test the construct validity, we assessed the relationship between the GAD-7 and the scores of socio-demographic and clinical variable. Moreover, to test the unidimensionality of GAD-7 an exploratory principal component factor analysis [34] was performed. Kaiser-Meyer-Olkin (KMO) measure of sampling adequacy and Bartlett’s test of sphericity were computed before the factor analysis.

To test the criterion validity GAD-7 scores were compared with the scores obtained by the other health status and quality of life measures. Concordances between criteria were computed by correlation coefficients and chi-square independence tests.

Besides these tests previously referred, we also performed descriptive analyses including measures of central tendency and dispersion.

This study followed the basic ethical principles set by the Declaration of Helsinki and has been approved by the Ethics Board of the Lisbon Psychiatric Hospital. All participants signed an informal consent, without any benefits. Data collection was anonymous, without any reference to patients personal identity, which was encoded in all study documents.

## Results

### The sample

Table 1 shows the distributions of the main socio-demographic and clinical variables.

**Table 1:** **Socio-demographic and clinical variables**

**Variable**	**Value**	**N**	**%**
Sample		100	100.0
Gender	Female	78	78.8
	Male	21	21.2
Age	Mean ± sd	52.2 ± 13.5	
	Min – Max	21 - 78	
Education	≤4 years	44	44.9
	5 to 9 years	22	22.4
	10-12 years	21	21.4
	>12 years	11	11.2
Family status	Single	11	11.0
	Married/Living together	38	68.0
	Divorced	17	17.0
	Widowed	4	4.0
Employment status	Employed	32	32.3
	Unemployed/Student	21	21.2
	Retired	40	40.4
	Sick leave	6	6.1
Clinical background	Yes	59	59.0
	No	41	41.0
Psychiatric background	Yes	80	80.8
	No	19	19.2
Physical symptoms ^1^	Pain	45	45.0
	Headache	43	43.0
	Tremors	32	32.0
	Palpitations	46	46.0
	Sudoresis	40	40.0
	Difficulty breathing	22	22.0
	Nausea	14	14.0
	Diarrhoea	13	13.0
	Other	2	2.0

sd: standard deviation.

Min-Max: Minimum-Maximum.

^1^A patient may have more than one symptom.

The sample included 78.8% of female with a mean age of 52.2 ± 13.5. Regarding the education, 44.9% had, at most, four years of schooling, 32.3% were employed and 68.0% were married or lived together. Among these patients, 59.0% had a previous clinical diagnosis and 80.8% a previous psychiatric diagnosis. In what concerns physical symptoms, the highest prevalent were palpitations, pain, headaches, and sudoresis.

Table 2 presents the distributions of the health status, symptoms and health-related quality of live variables.

**Table 2:** **Quality of life variables**

**Variable**	**Dimension**		**N**	**%**
GAD-7	Index	Normal	4	4.0
		Mild anxiety	7	7.1
		Moderate anxiety	18	18.2
		Severe anxiety	70	70.7
		Mean ± sd	15.7 ± 4.6	
		Min - Max	2 - 21	
EQ-5D	Index	[−0.50; −0.25]	2	2.0
		[−0.25; 0.00]	1	1.0
		[ 0.00; +0.25]	17	17.2
		[+0.25; +0.50]	33	33.3
		[+0.50; +0.75]	27	27.3
		[+0.75; +1.00]	19	19.2
		Mean ± sd	0.46 ± 0.29	
		Min - Max	−0.37 - 1.00	
	VAS	[ 0; 25]	17	17.9
		[25; 50]	29	30.5
		[50; 75]	40	42.1
		[75; 100]	9	9.5
		Mean ± sd	44.33 ± 22.27	
		Min - Max	0 - 95	
HADS	Anxiety	Without symptoms	12	12.0
		With symptoms	88	88.0
		Mean ± sd	13.6 ± 4.2	
		Min - Max	1 - 21	
	Depression	Without symptoms	31	31.0
		With symptoms Mean	69	69.0
		Mean ± sd	10.8 ± 4.4	
		Min - Max	0 - 21	
STAI	State Anxiety	Mean ± sd	48.5 ± 4.3	
		Min - Max	39 - 61	
	Trait Anxiety	Mean ± sd	50.5 ± 4.9	
		Min - Max	38 - 60	

sd: standard deviation Min/Max: Minimum/Maximum.

Regarding the health status and quality of life, the majority of the patients (70.7%) may be classified as severely anxious, which is evidenced by the self-perception given by the EQ-5D: mean index = 0.46 and mean VAS = 44.33. More than four fifth of the patients (88.0%) had symptoms of anxiety and 69.0% showed symptoms of depression. Accordingly, both the state and trait anxiety scores were median, indicating a moderate form of anxiety.

### Feasibility

The mean GAD-7 completion time was 2.3 ± 1.3 minutes, ranging from 30 seconds to 4.7 minutes. All items were filled. To assess the floor and ceiling effects of GAD-7 we analysed the distribution of each item (see Table 3).

**Table 3:** **Distribution of GAD-7 items**

**GAD-7 item**	**Not at all**	**Several days**	**More than half the days**	**Nearly every day**
Feeling nervous, anxious or on edge	3.0%	10.1%	33.3%	53.5%
Not being able to stop or control worrying	1.0%	17.2%	24.2%	57.6%
Worrying too much about different things	2.0%	12.1%	33.3%	52.5%
Trouble relaxing	5.1%	12.1%	38.4%	44.4%
Being so restless that it is hard to sit still	8.1%	19.2%	34.3%	38.3%
Becoming easily annoyed or irritable	3.0%	17.2%	30.3%	49.5%
Feeling afraid as if something awful might happen	9.1%	13.1%	37.4%	40.4%

No major floor effect was found. In fact, only items 5 and 7 had a percentage higher than 8%. On the other hand, our sample showed a group of patients with very severe levels of anxiety.

### Reliability

The Cronbach’s α obtained for the GAD’s seven items was an excellent value (0.880) and it maintains excellent even if we delete an item, as shown in the second column of Table 4.

**Table 4:** **Reliability indicators**

**GAD-7 item**	**α If item deleted**	**Item-total correlation**	**Test-retest correlation coefficient**	**Intraclass correlation**
Feeling nervous, anxious or on edge	0.854	0.817**	0.857**	0.819
Not being able to stop or control worrying	0.879	0.650**	0.576**	0.570
Worrying too much about different things	0.863	0.760**	0.677**	0.671
Trouble relaxing	0.852	0.828**	0.596**	0.555
Being so restless that it is hard to sit still	0.860	0.790**	0.629**	0.620
Becoming easily annoyed or irritable	0.867	0.739**	0.654**	0.644
Feeling afraid as if something awful might happen	0.865	0.764**	0.931**	0.930

**p < 0.01.

Moreover, all items showed high item-total correlation scores (column 3) and high test-retest correlation coefficients (column 4) and intraclass correlation coefficients (column 5).

### Construct validity

Table 5 shows the sensitivity of GAD-7 index over the different values of the socio-demographic and clinical variables.

**Table 5:** **Relationship between GAD-7 index and socio-demographic and clinical variables**

**Variable**	**Value**	**GAD-7 index**	**t/F**	**Sig**
Gender	Female	15.96	1.14	0.256
	Male	14.65		
Age	Less than 40 years	13.41	3.861	0.024
	Between 40 and 59 years	16.45		
	60 or more years	16.30		
Education	≤4 years	17.45	7.631	0.001
	5 to 9 years	15.05		
	≥10 years	13.62		
Family status	Married/Living together	15.84	0.340	0.734
	Not married	15.50		
Employment status	Employed	13.56	−0.3660	0.000
	Non-employed	16.91		
Clinical background	Yes	16.20	1.262	0.210
	No	15.02		
Psychiatric background	Yes	15.90	0.542	0.589
	No	15.26		
Pain	Yes	17.33	3.514	0.001
	No	14.39		
Headache	Yes	17.02	2.664	0.009
	No	14.73		
Tremors	Yes	17.84	4.054	0.000
	No	14.72		
Palpitations	Yes	16.78	2.181	0.032
	No	14.81		
Sudoresis	Yes	17.15	2.625	0.010
	No	14.76		
Difficulty breathing	Yes	18.09	2.848	0.005
	No	15.05		
Nausea	Yes	18.21	2.242	0.027
	No	15.32		
Diarrhea	Yes	18.46	3.577	0.001
	No	15.31		

t: Student’s t.

F: Fisher’s F.

Sig: significance (p-value).

As we can draw from this table there is no significant difference of GAD-7 index regarding gender, family status, and clinical or psychiatric background. We also evidenced significant lower GAD-7 index, i.e., better health, for younger (less than 40 years old) patients, those with higher education, employed and without symptoms.

To test the construct validity we also performed a principal component factor analysis and we evidenced the desirable unidimensional structure, corresponding to 58.8% of explained variance.

GAD-7 index was correlated to the other health outcome indices. Starting with EQ-5D, both indices had a significant correlation (EQ-5D index: −0.538; VAS: −0.378). However, the correlations with STAI indices were also smaller, although significant (S-Anxiety: r = 0.378; T-Anxiety: r = 0.353). In what concerns the HADS, GAD-7 showed to be better correlated with anxiety subscale (r = 0.699) than with depression subscale (r = 0.450).

Comparing the anxiety classification obtained by GAD-7, HADS-Anxiety and HAD-Depression measures we noticed that they are not independent, meaning that patients without symptoms revealed by both HADS indices are also classified by GAD-7 as normal or with a mild anxiety. The corresponding chi-squared values (HADS-A: χ^2^ = 43.59; HADS-D: χ^2^ = 27.73) ware associated to p-values lower than 0.005.

On the other hand patients with symptoms detected by HADS have always a higher GAD-7 index, as shown in Table 6.

**Table 6:** **Comparison between GAD-7 index with HADS**

**Variable**	**Value**	**GAD-7 index**	**t**	**Sig**
HADS – Anxiety	Without symptoms	8.42	−7.329	0.000
	With symptoms	16.74		
HADS – Depression	Without symptoms	12.17	−5.937	0.000
	With symptoms	17.27		

t: Student’s t.

Sig: significance (p-value).

## Discussion

The authors intended to test the culturally adapted into Portuguese version of the GAD-7 scale concerning feasibility, reliability and validity.

Excellent reliability values were found when comparing each item of the measure with the total scores and also in the test-retest, showing an excellent homogeneity in concept measurement and stability between evaluations over time.

We correlated the scores from GAD-7 with those from HADS. As a result, we evidenced a very high significant correlations with both HADS anxiety and depression subscales, which supports the use of the GAD-7 as a screening tool. However, regarding the STAI a significant but weaker correlation was found probably due its complexity.

When comparing the GAD-7 results with other health outcome measures yielded from the EQ-5D scale, a relation was found between GAD assessments using the scale and the disability level assessed by several domains of daily life, which is in accordance with previous data reported in GAD studies [35-37]. This shows that this instrument is a solid tool for easily exploring patients with GAD, establishing the level of severity, and linking it to the degree of disability in the main areas of daily living. Thus, GAD emerges as a strong predictor of functional impairment [19].

The results obtained for the Portuguese version of the GAD-7 and the impact of the socio-demographic characteristics and clinical variables in the measurements were in line with data available in the reviewed literature, with exception of family status and clinical/psychiatric background. In our sample, anxiety levels were independent from family status, with no significant difference between single, married/living together, divorced and widowed. Another unexpected result was that clinical and psychiatric background did not influence the results. This might be due to the fact that the sample was merely clinical and no general population controls were used.

Our study showed some limitations worth reviewing. It was conducted in only one major psychiatric hospital. However the authors consider it to be representative of the existing linguistic variability in the country and, therefore, sufficiently representative of the whole national territory. Additionally, a sample size of 100 patients and the absence of a control group may have conditioned some results.

Another possible limitation might be the fact that psychiatric diagnoses was performed through psychiatric interview on clinical bases. Moreover, our sample contained exclusively participants with a GAD diagnosis, which may lead to a variance reduction and a risk of underestimated correlation coefficients.

Despite the limitations, the good performance of the scale adapted into Portuguese, its short administration time and highly cost-effective administration make the GAD-7 a useful tool for standard clinical practice for patient screening purposes.

## Conclusion

The GAD-7 scale has shown to be highly correlated not only with specific anxiety measures but also with disability measures showing excellent psychometric properties, high discriminant ability, briefness, and fast administration. For these reasons, the successful validation into Portuguese of the GAD-7 scale, will allow an increasingly early detection and treatment of these patients, thus improving their quality of life and reducing medical and psychiatric complications.
